# Supplementary material for: Integrated metabolomic and transcriptomic analysis reveal the effect of mechanical stress on sugar metabolism in tea leaves (Camellia sinensis) post-harvest
Source: PeerJ. 2023 Feb 8;11:e14869. doi: 10.7717/peerj.14869 (PMC9921968; doi:10.7717/peerj.14869)
Supplement: Supplemental Information 1 [file peerj-11-14869-s001.zip › Supplemental Files/Fig S1-S9/Supplemental Figure legends.docx]

Figure S1. Validation of the transcriptome by qRT-PCR. Thirteen DEGs were selected for qRT-PCR analysis using CsGADPH as a control. The bars represent the qRT-PCR results, while the line represents the RNA-seq results. Figure S2. TIC of quality control sample in negative mode, as revealed by mass spectrometry detection. Figure S3. TIC of C0 sample in negative mode, as revealed by mass spectrometry detection. Figure S4. TIC of C15 sample in negative mode, as revealed by mass spectrometry detection. Figure S5. TIC of V15 sample in negative mode, as revealed by mass spectrometry detection. Figure S6. TIC of quality control sample in positive mode, as revealed by mass spectrometry detection. Figure S7. TIC of C0 sample in positive mode, as revealed by mass spectrometry detection. Figure S8. TIC of C15 sample in positive mode, as revealed by mass spectrometry detection. Figure S9. TIC of V15 sample in positive mode, as revealed by mass spectrometry detection.
