# Supplementary material for: Integrated metabolomic and transcriptomic analysis reveal the effect of mechanical stress on sugar metabolism in tea leaves (Camellia sinensis) post-harvest
Source: PeerJ. 2023 Feb 8;11:e14869. doi: 10.7717/peerj.14869 (PMC9921968; doi:10.7717/peerj.14869)
Supplement: Supplemental Information 1 [file peerj-11-14869-s001.zip › Supplemental Files/Table S1.docx]

Table S1 Primers used for qRT-PCR analysis

| Genes | Forword primer 5'-3' | Reverse primer 5'-3' |
| --- | --- | --- |
| TEA030570 | GCAATGGATGCTGAGGCAAT | TTTTCCCTGTCGCAGTGTCA |
| TEA009994 | TCTCCATCGCCGAGTTCTCT | GCAAGTCAAGCCCCAGAAGT |
| TEA032228 | GGAGCAATGCCTCTGGTTCT | GCAAGTTTGGGATCTGCTGC |
| TEA028940 | TCGATGACCAGCAGAAGTCG | CCATGAAGGTCCAGGAGTGC |
| TEA001334 | ACTGGTGGTTATGGCAGCGT | CAAGCTTCCCCTTTCCATGT |
| TEA003353 | CTTGGTGCACAATCCACACG | AGATGAGCCCCTTTGCTGAC |
| TEA012904 | CTCGGCTGGACCGACTCGAAC | GAGATACTGGAGCGAGTTCAA |
| TEA000370 | TCCAAGGCATGCCTGGAACT | CCGCTTCACCAAGAGTGCAT |
| TEA023566 | CTGGATCTGGTCTGGATCGA | GCATCTCCATGTCGCCAATG |
| TEA017924 | GTGGGGACTAGTACAGATCA | GTTCCCATGCTTGACAAGGTG |
| TEA017875 | GGATGGCACGTCATGGACGC | GAGTTAGACGTCACTTTGG |
| TEA004678 | GGGTCCGAATGAGAAACTGG | GGCTTGGACGTCTCGGGGAG |
