# Supplementary figures and images for: Integrated metabolomic and transcriptomic analysis reveal the effect of mechanical stress on sugar metabolism in tea leaves (Camellia sinensis) post-harvest
Source: PeerJ. 2023 Feb 8;11:e14869. doi: 10.7717/peerj.14869 (PMC9921968; doi:10.7717/peerj.14869)

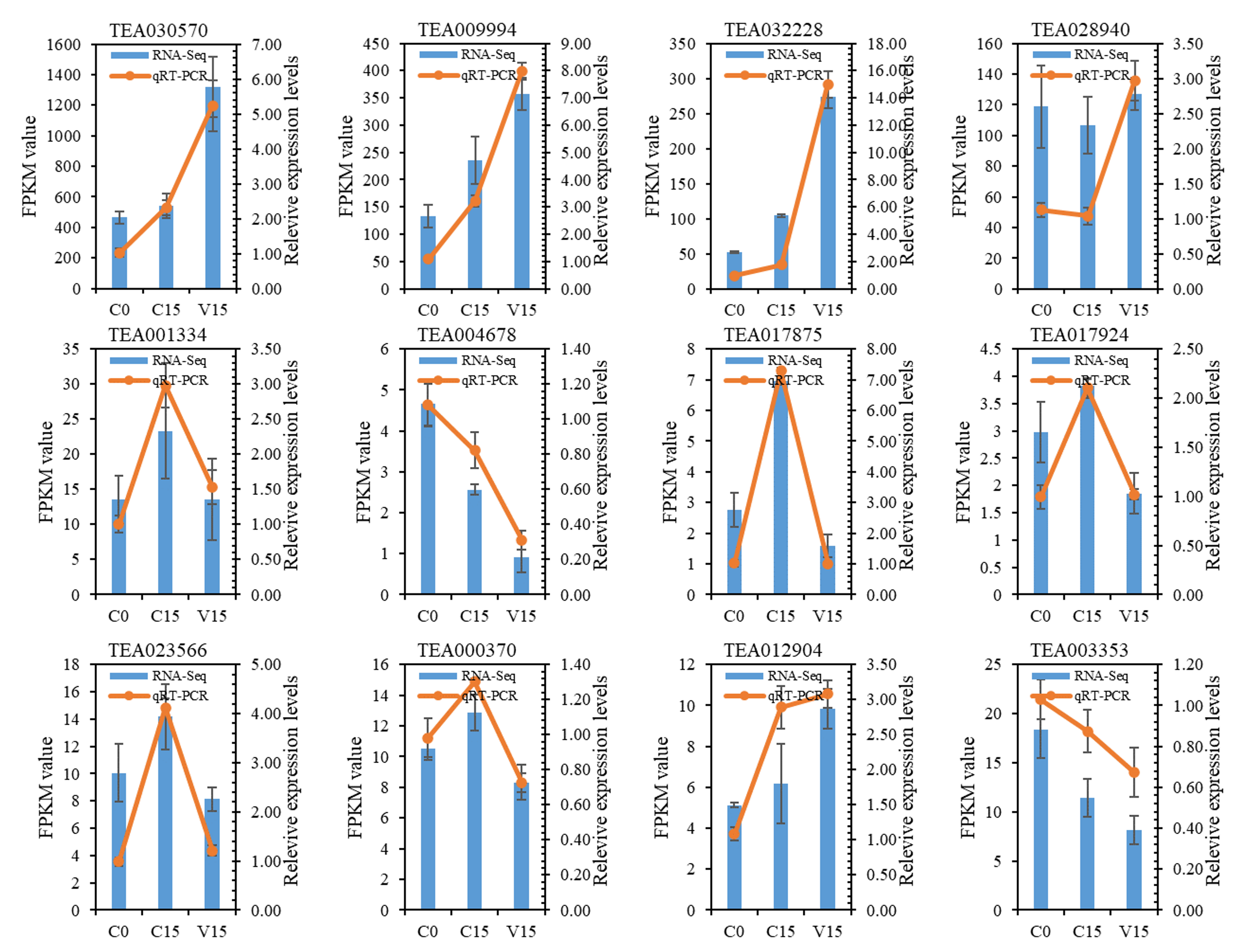

Supplement: Supplemental Information 1 [file peerj-11-14869-s001.zip › Supplemental Files/Fig S1-S9/Fig. S1.png]

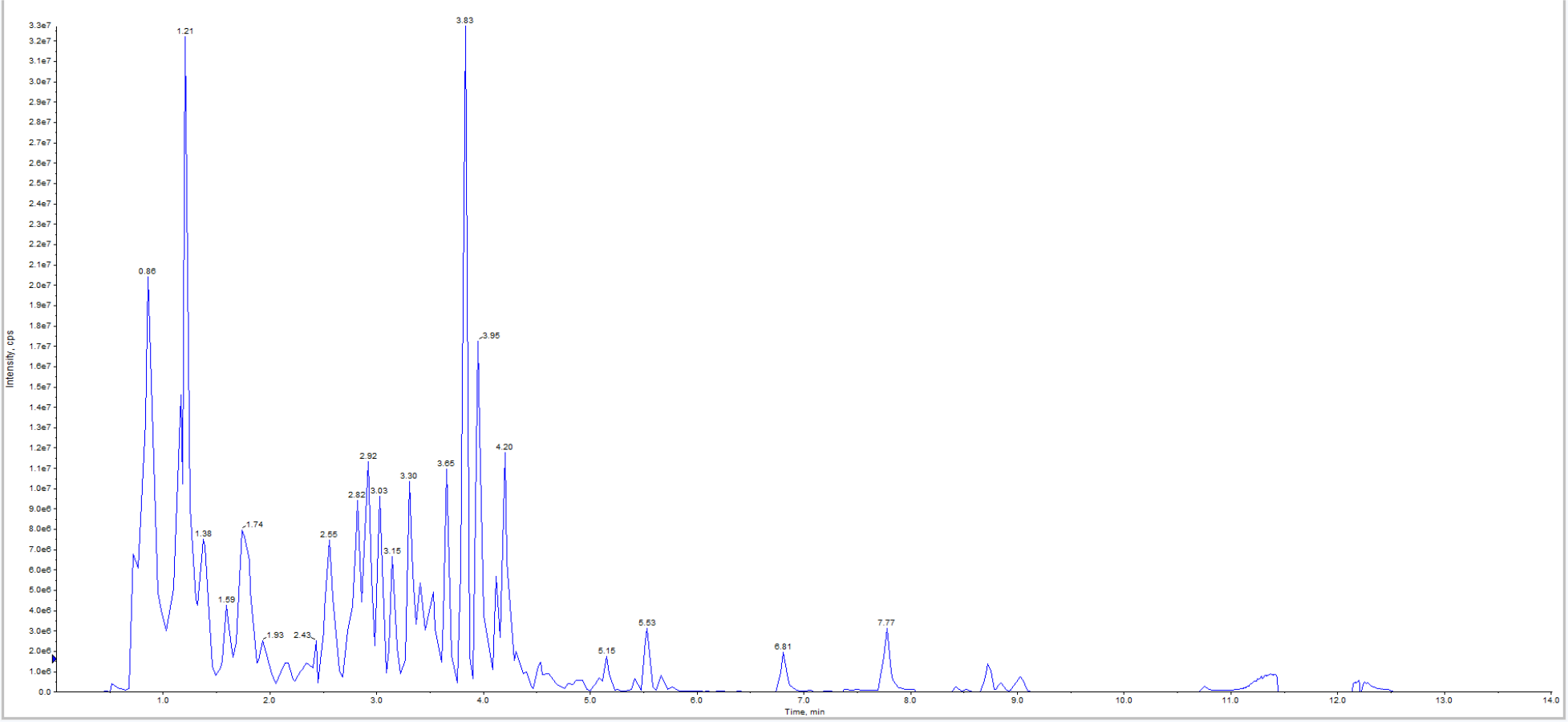

Supplement: Supplemental Information 1 [file peerj-11-14869-s001.zip › Supplemental Files/Fig S1-S9/Fig. S2 TIC of QC sample in negative mode, as revealed by mass spectrometry detection.png]

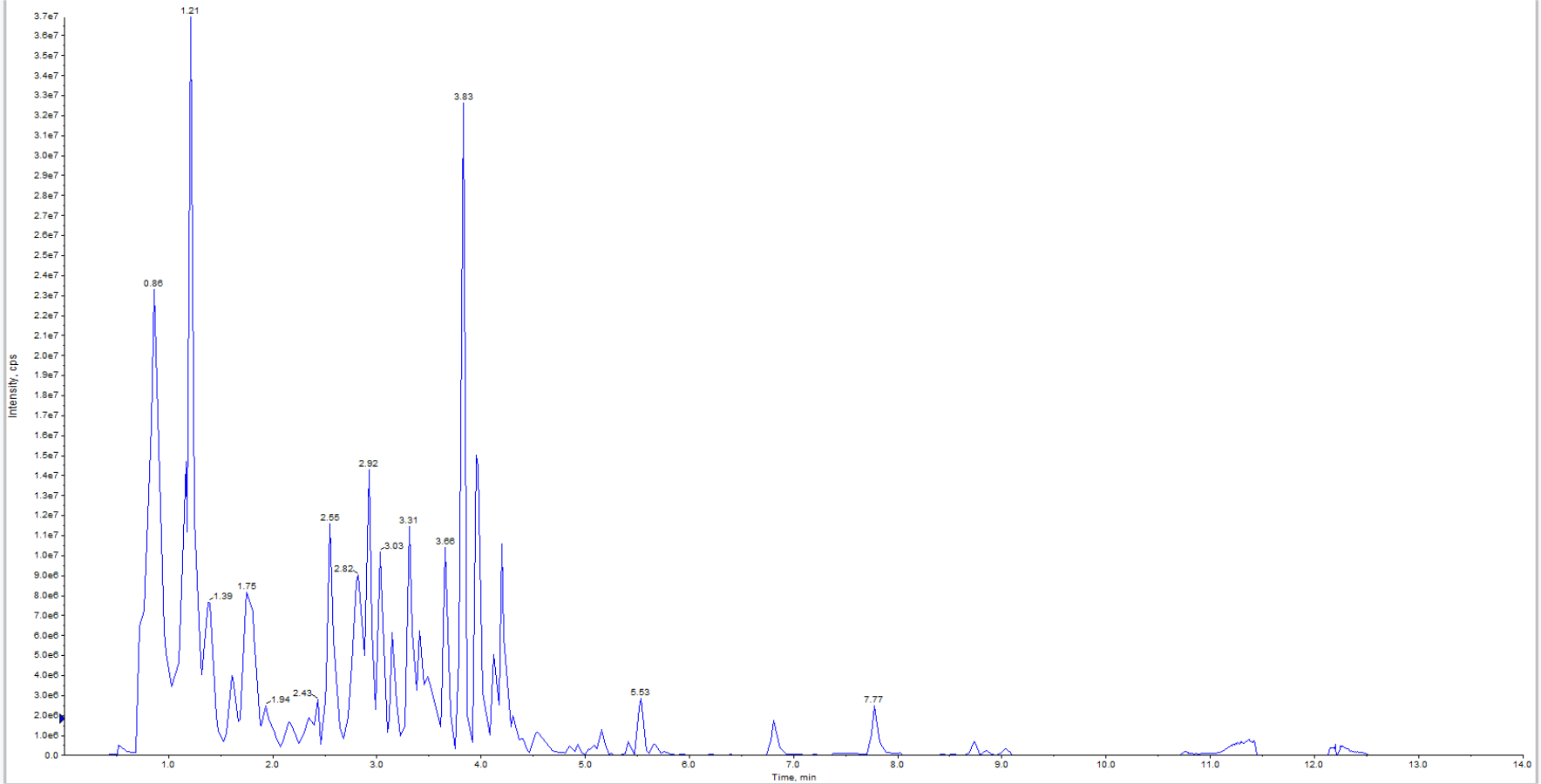

Supplement: Supplemental Information 1 [file peerj-11-14869-s001.zip › Supplemental Files/Fig S1-S9/Fig. S3 TIC of C0 sample in negative mode, as revealed by mass spectrometry detection.png]

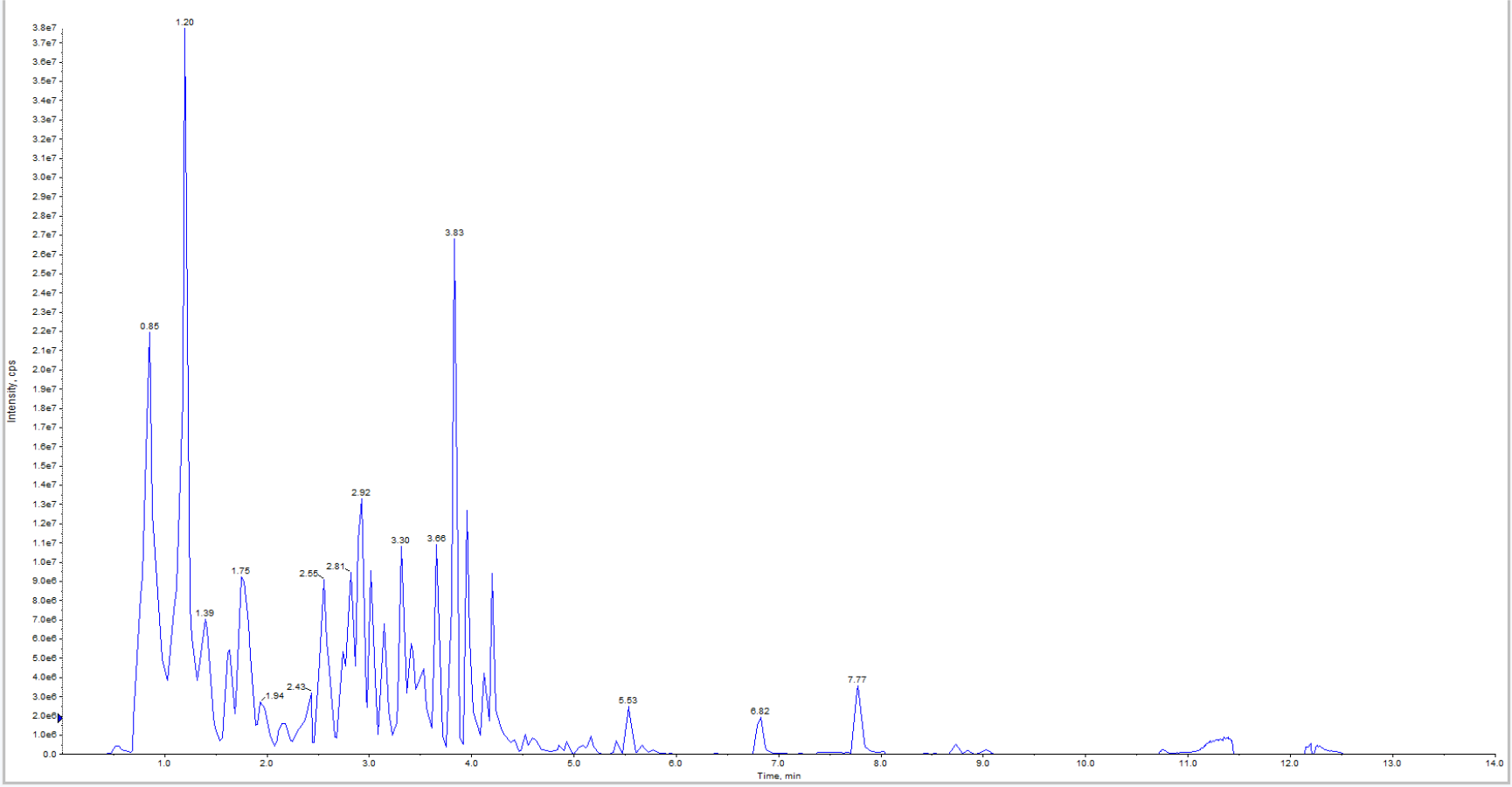

Supplement: Supplemental Information 1 [file peerj-11-14869-s001.zip › Supplemental Files/Fig S1-S9/Fig. S4 TIC of C15 sample in negative mode, as revealed by mass spectrometry detection.png]

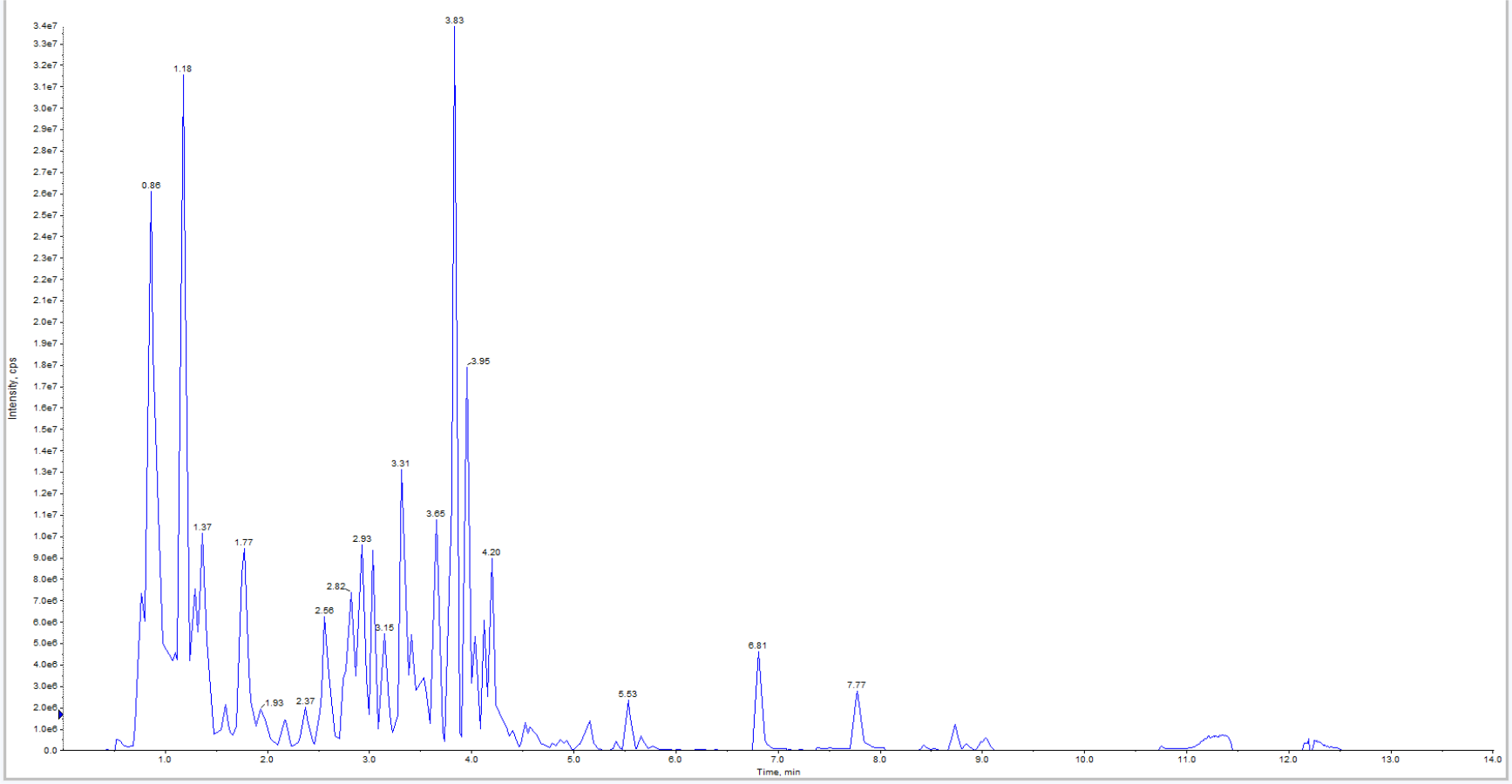

Supplement: Supplemental Information 1 [file peerj-11-14869-s001.zip › Supplemental Files/Fig S1-S9/Fig. S5 TIC of C15 sample in negative mode, as revealed by mass spectrometry detection.png]

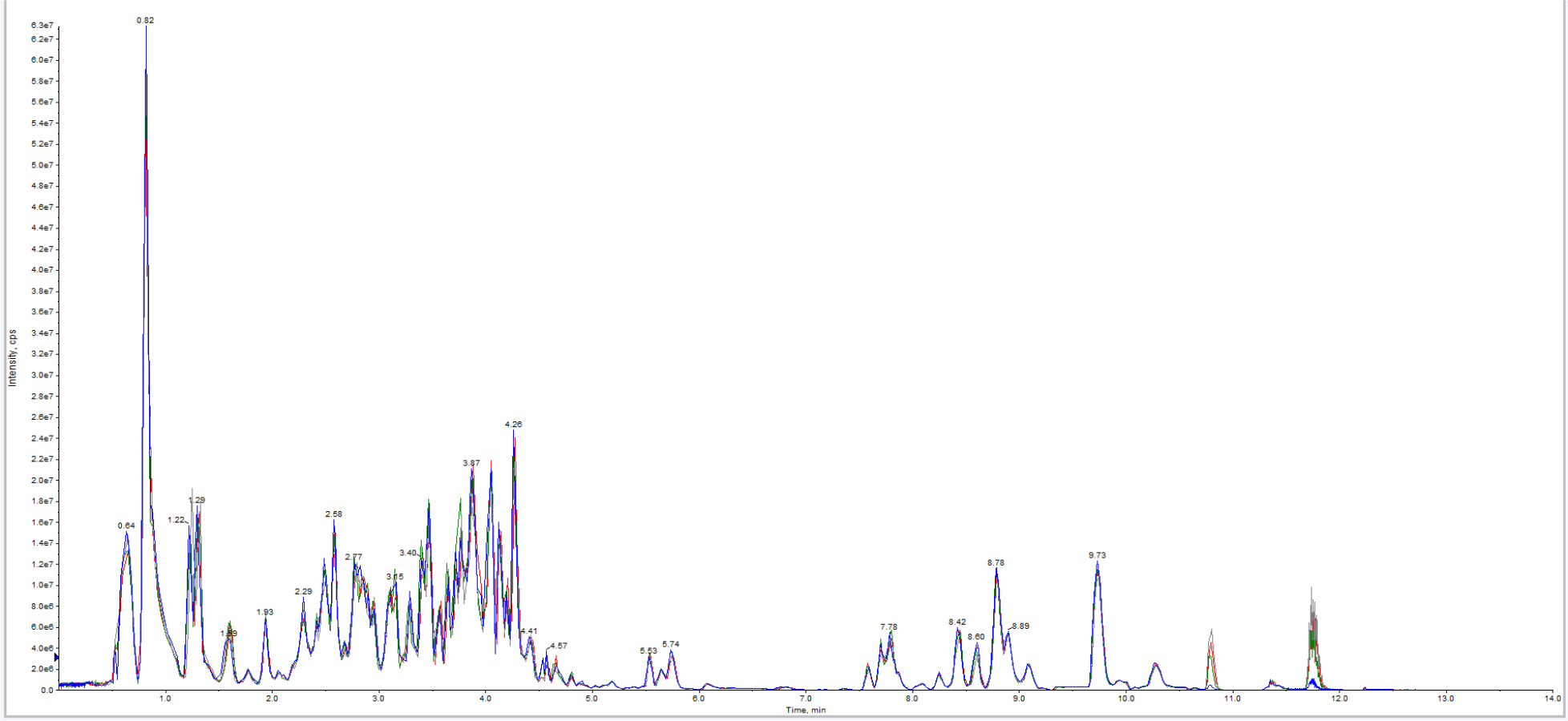

Supplement: Supplemental Information 1 [file peerj-11-14869-s001.zip › Supplemental Files/Fig S1-S9/Fig. S6 TIC of quality control sample in positive mode, as revealed by mass spectrometry detection.png]

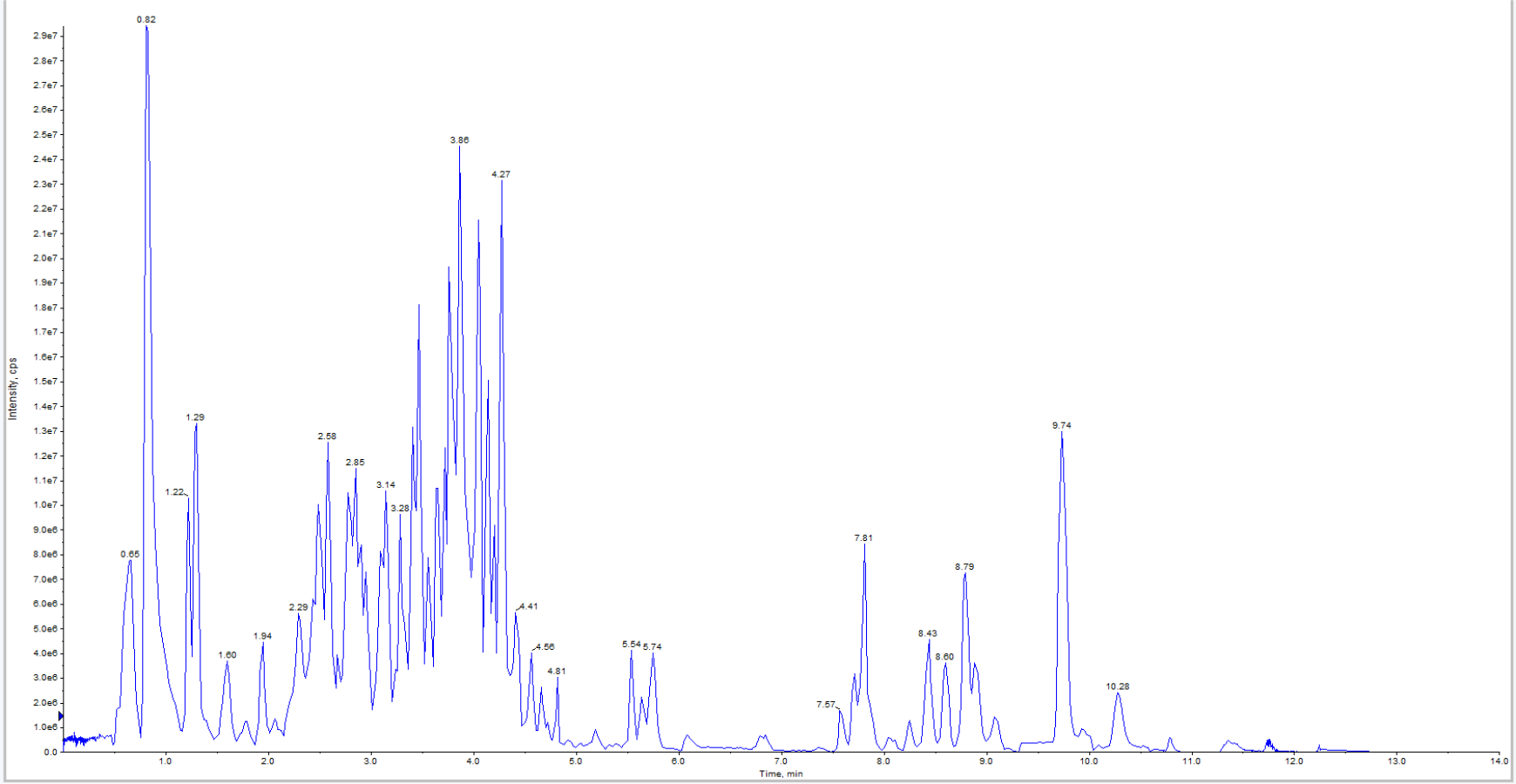

Supplement: Supplemental Information 1 [file peerj-11-14869-s001.zip › Supplemental Files/Fig S1-S9/Fig. S7 TIC of C0 sample in positive mode, as revealed by mass spectrometry detection.png]

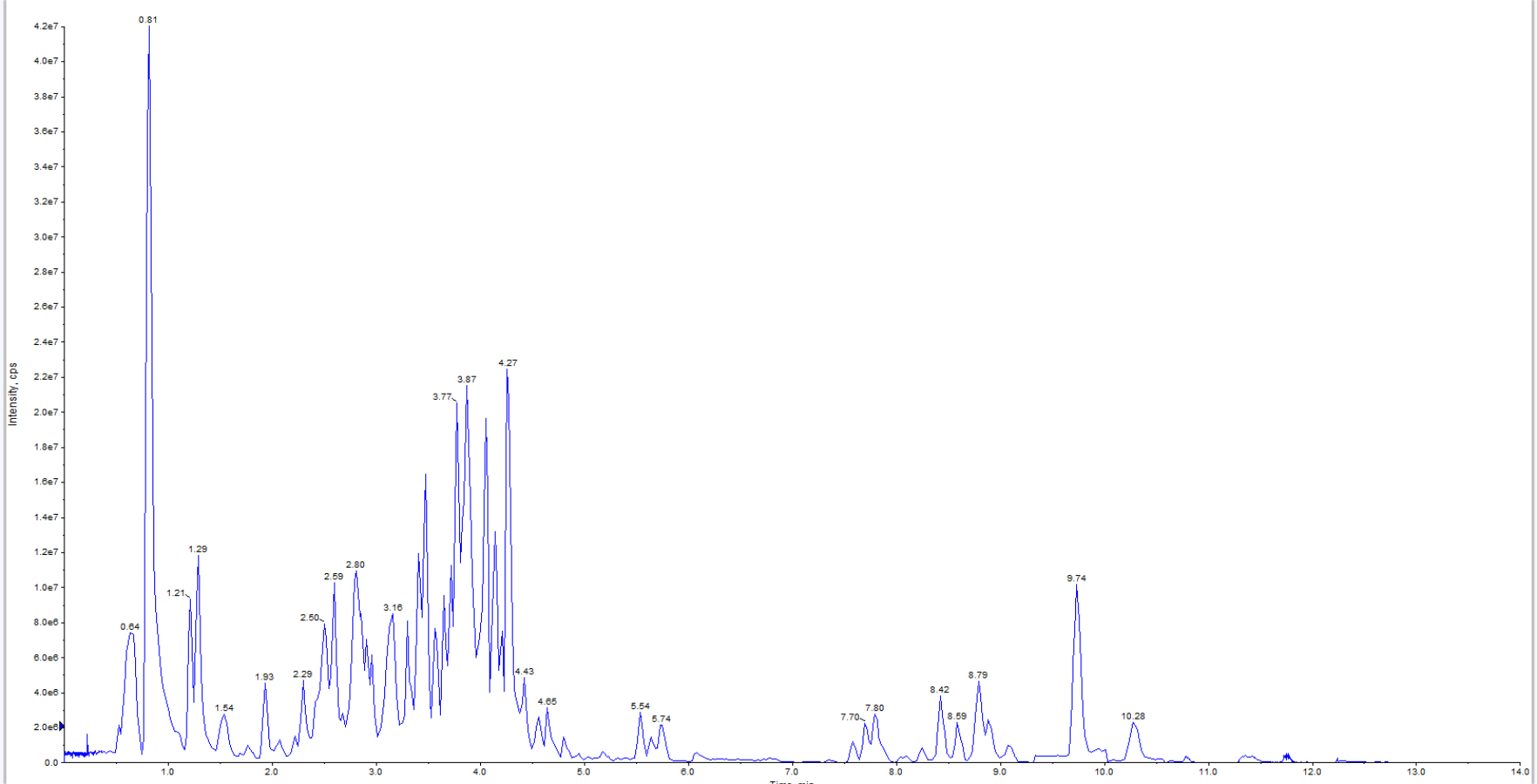

Supplement: Supplemental Information 1 [file peerj-11-14869-s001.zip › Supplemental Files/Fig S1-S9/Fig. S8 TIC of C15 sample in positive mode, as revealed by mass spectrometry detection.png]

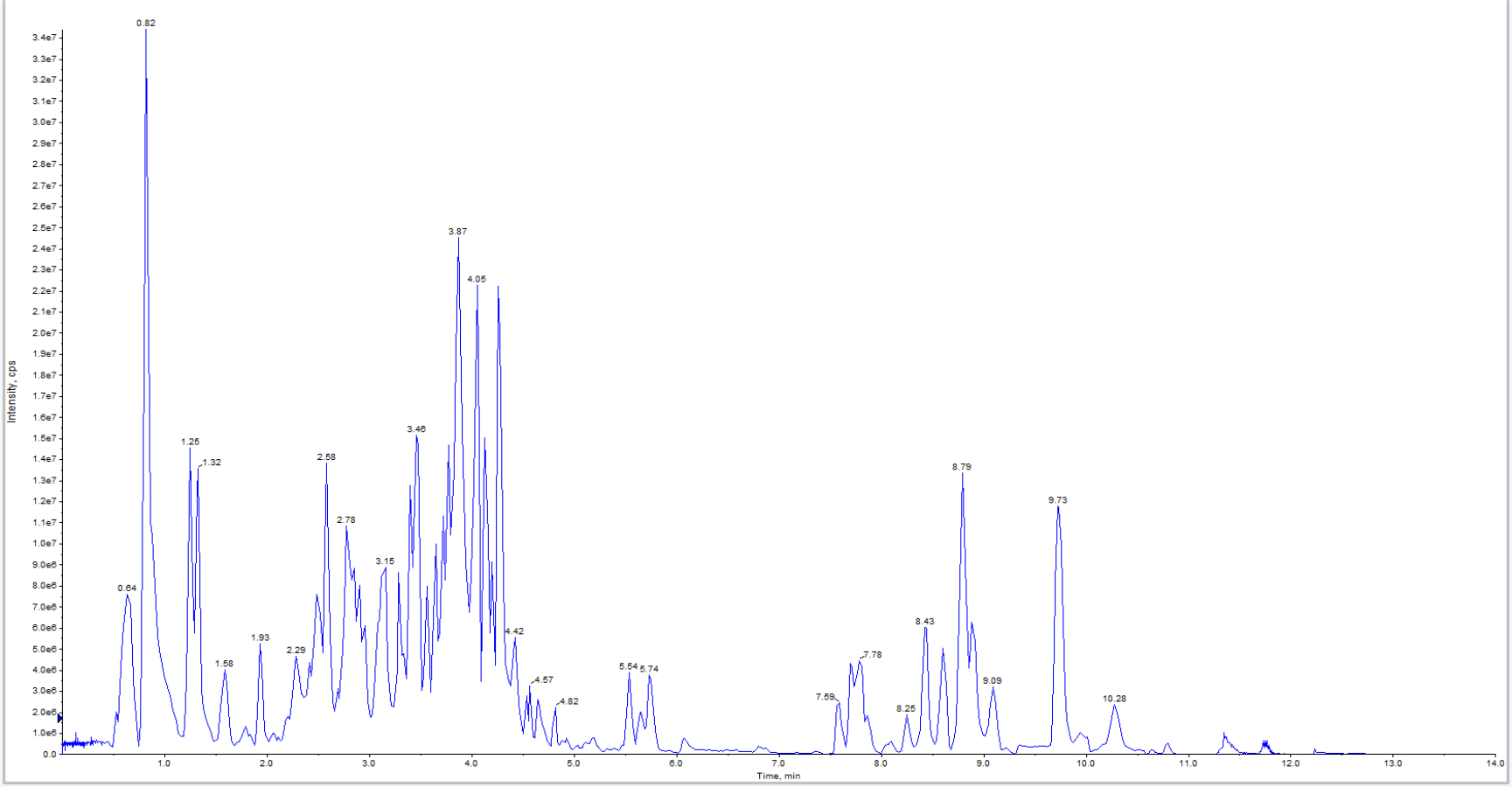

Supplement: Supplemental Information 1 [file peerj-11-14869-s001.zip › Supplemental Files/Fig S1-S9/Fig. S9 TIC of V15 sample in positive mode, as revealed by mass spectrometry detection.png]
